# Burnout, Depression, and Borderline Personality: A 1,163-Participant Study

**DOI:** 10.3389/fpsyg.2017.02336

**Published:** 2018-01-11

**Authors:** Renzo Bianchi, Jean-Pierre Rolland, Jesús F. Salgado

**Affiliations:** ^1^University of Neuchâtel, Neuchâtel, Switzerland; ^2^Université Paris Nanterre, Nanterre, France; ^3^Universidade de Santiago de Compostela, Santiago de Compostela, Spain

**Keywords:** affect, borderline personality, burnout, depression, mood, neuroticism, occupational stress

## Abstract

We examined the association of burnout with borderline personality (BP) traits in a study of 1,163 educational staff (80.9% women; mean age: 42.96). Because burnout has been found to overlap with depression, parallel analyses of burnout and depression were conducted. Burnout symptoms were assessed with the Shirom-Melamed Burnout Measure, depressive symptoms with the PHQ-9, and BP traits with the Borderline Personality Questionnaire. Burnout was found to be associated with BP traits, controlling for neuroticism and history of depressive disorders. In women, burnout was linked to both the “affective insecurity” and the “impulsiveness” component of BP. In men, only the link between burnout and “affective insecurity” reached statistical significance. Compared to participants with “low” BP scores, participants with “high” BP scores reported more burnout symptoms, depressive symptoms, neuroticism, and occupational stress and less satisfaction with life. Disattenuated correlations between burnout and depression were close to 1, among both women (0.91) and men (0.94). The patterns of association of burnout and depression with the main study variables were similar, pointing to overlapping nomological networks. Burnout symptoms were only partly attributed to work by our participants. Our findings suggest that burnout is associated with BP traits through burnout-depression overlap.

## Introduction

Burnout has been conceived of as a syndrome combining physical fatigue, cognitive weariness, and emotional exhaustion and resulting from chronic, unresolvable stress at work (Shirom and Melamed, 2006). The job-induced character of burnout has been considered a key distinguishing characteristic of the syndrome (Schaufeli and Enzmann, 1998; Maslach et al., 2001; Shirom, 2005). Burnout has been associated with a variety of adverse health outcomes, for instance, coronary heart disease (Toker et al., 2012). Although not considered a nosological entity in the latest editions of the *Diagnostic and Statistical Manual of Mental Disorders* (DSM-5; APA, 2013) and the *International Classification of Diseases* (ICD-10; World Health Organization, 2010), burnout has elicited growing interest among the psychology and the psychiatry community over the last decades.

Borderline personality (BP) is characterized by hypersensitivity to rejection and fear of abandonment, impulsivity (e.g., substance abuse, binge eating), identity disturbance (i.e., markedly and persistently unstable self-image or sense of self), recurrent self-destructive or suicidal ideation/behaviors, affective instability (e.g., intense episodic dysphoria or anxiety), chronic feelings of emptiness, angry outbursts, and transient, stress-related paranoid thoughts or dissociative symptoms (Gunderson, 2011; APA, 2013). BP disorder has been found to have a prevalence of about 3–6% in the US general population—depending on the employed diagnostic algorithms—and to be associated with considerable mental and physical disability, especially among women (Grant et al., 2008; Trull et al., 2010).

BP disorder notably shows pronounced comorbidity with major and persistent depressive disorders (Köhling et al., 2015; Bornovalova et al., 2017; Witt et al., 2017). While BP has been associated with general interpersonal dysfunction (Wilson et al., 2017), it has been linked to enhanced sensitivity to the mental states of others (Fertuck et al., 2009; Trull and Widiger, 2013). Individuals with BP tend to experience impairment at work and to have tumultuous employment histories (Skodol et al., 2002; Trull et al., 2010; Wille et al., 2013). However, factors such as parental socioeconomic status, level of education, and intellectual quotient appear to play a compensatory role and to allow individuals with BP to reach high-income positions (Deary et al., 2005; Niesten et al., 2016).

It has been emphasized in recent years that personality pathology should be apprehended not only categorically but also dimensionally (Widiger and Trull, 2007; Trull and Widiger, 2013; Tyrer et al., 2015; Hengartner and Lehmann, 2017; Horwood and Anglim, 2017; Witt et al., 2017). Both personality disorders and “personality difficulties” (i.e., subclinical traits) have been longitudinally linked to the occurrence of mental health and social problems (Moran et al., 2016). Such links have been specifically established in BP research (e.g., ten Have et al., 2016).

While burnout has been assumed to result from a misfit between the resources and expectations of the individual on the one hand, and the demands and realities of his/her work on the other hand (Schaufeli and Enzmann, 1998), burnout researchers have paid more attention to the environmental/external, than to the dispositional/internal, contributory factors to the syndrome (Swider and Zimmerman, 2010; Bianchi and Schonfeld, 2016). Burnout has nevertheless been linked to dispositional/internal contributors such as pathological perfectionism and need for approval, ruminative responses, pessimistic attributions, neuroticism, extraversion, negative affectivity, and positive affectivity (Alarcon et al., 2009; Bianchi and Schonfeld, 2016). To date, there has been a paucity of research on the relationship between burnout and BP^1^. Because of its very features, BP is likely to bear heavily on (a) the frequency and intensity of stressful experiences and (b) the ability of the individual to successfully cope with the encountered stressors. Recent empirical research consistently supports such a view (Jovev and Jackson, 2006; Powers et al., 2013; Wille et al., 2013; Aleknaviciute et al., 2016). In occupational contexts, the stormy interpersonal relationships fostered by BP (Wilson et al., 2017), for example, may be reflected in stressogenic conflicts with colleagues or superiors. All in all, BP may thus be a vulnerability factor for stress-related syndromes such as burnout.

The main aim of the present study was to examine the association between burnout and BP traits. In light of the links established in past research between BP and stress on the one hand, and stress and burnout on the other hand, we hypothesized that BP would be positively associated with burnout. Because some features of BP may overlap with characteristics of neuroticism (e.g., dysphoria, anxiety, anger), we controlled for neuroticism in our analyses of this association. In addition, because burnout has been found to overlap with depression (Taris, 2006; Ahola et al., 2014; Schonfeld and Bianchi, 2016; Bianchi et al., 2017e,f), we conducted comparative analyses of burnout and depression in this study. We notably examined whether burnout and depression showed different patterns of association with BP traits. Examining the association between burnout and BP traits is important for at least two reasons. First, such an examination can help restore balance in research on the environmental/external vs. dispositional/internal contributory factors to burnout. Second, research on BP in the occupational context remains limited. Investigating the relationship between burnout and BP traits may allow us to better understand why individuals with BP are more likely to experience unemployment, frequent job changes, or periods of disability (Skodol et al., 2002).

## Methods

### Participants and data collection

School administrators were contacted by email in November and December 2016 in two French geographic areas. They were asked to respond to an Internet survey and to transmit the survey in question to the other educational staff working in their schools (e.g., teachers, administrative assistants, bursars), to offer them the opportunity to respond as well. Online questionnaires have been shown to be as reliable and valid as traditional, paper-and-pencil questionnaires (see Bianchi et al., 2016b). The survey included measures of burnout, depression, BP traits, neuroticism, life satisfaction, occupational stress as well as a sociodemographic and health questionnaire. Respondents were informed that, by completing the survey, they were giving consent to their inclusion in the study. Two points were emphasized in pre-survey instructions: first, that participation was entirely voluntary; second, that respondents could stop completing the survey at any moment and for any reason should they so choose. The only eligibility criterion for taking part in the study was to be currently employed as an educational staff member in an elementary school, a middle school, or a high school. A total of 1,163 educational staff (80.9% women; mean age: 42.96) completed the survey. A vast majority of the recruited participants were teachers, a population thought to be particularly affected by job stress and burnout (Maslach et al., 2001). Because our recruitment procedure did not allow us to know how many workers got access to our survey through school administrators' relay, the response rate to our survey could not be estimated.

### Measures

#### Burnout and depression

Burnout symptoms were assessed with the 14-item version of the Shirom-Melamed Burnout Inventory (SMBM; Cronbach's α = 0.95; Shirom and Melamed, 2006; Toker et al., 2012). The SMBM includes items related to physical fatigue (e.g., “I feel physically drained”), cognitive weariness (e.g., “My thinking process is slow”), and emotional exhaustion (e.g., “I feel I am unable to be sensitive to the needs of coworkers and recipients”). The SMBM has shown satisfactory psychometric properties, notably in its French version (see Sassi and Neveu, 2010; see also Bianchi and Brisson, 2017), and is one of the most widely used measures of burnout (e.g., Toker et al., 2012). By contrast with measures such as the Maslach Burnout Inventory (MBI; Maslach et al., 2016), the SMBM is in the public domain and reflects a view of burnout that is conceptually homogeneous (Shirom, 2005; Shirom and Melamed, 2006; Brisson and Bianchi, 2017). The instructions to respondents of the SMBM explicitly mention “work” as the context of reference of the questionnaire.

Depressive symptoms were assessed with the PHQ-9 (Cronbach's α = 0.86; Kroenke et al., 2001). The PHQ-9 is an increasingly-employed measure of depressive symptoms that has the advantage of directly targeting the nine diagnostic criteria for major depressive disorder of the DSM-5 (Kroenke et al., 2001; APA, 2013). The PHQ-9 relies on a 4-point scale, from 0 for *not at all*, to 3 for *nearly every day*. The respondent reports the symptoms he/she has experienced over the past 2 weeks. The PHQ-9 can be divided into two subscales (Beard et al., 2016; Martin-Subero et al., 2017), one involving the “affective-cognitive” symptoms of depression (items 1 [anhedonia], 2 [depressed mood], 6 [guilt/worthlessness], and 9 [suicidal ideation]; Cronbach's α = 0.79), and another involving the “somatic” symptoms of depression (items 3 [sleep disturbance], 4 [fatigue and loss of energy], 5 [appetite alteration], 7 [concentration impairment], and 8 [psychomotor malfunction]; Cronbach's α = 0.78). The psychometric and screening properties of the PHQ-9 have been found to be satisfactory in both patient and nonpatient samples (e.g., Kroenke et al., 2001; Martin et al., 2006; Beard et al., 2016; Martin-Subero et al., 2017). In a recent study of measurement equivalence, Arthurs et al. (2012) found evidence that the PHQ-9 could be used without adjustment in its English and French versions.

With the goal of enhancing the validity of the burnout-depression comparison, we standardized the time window of the two entities' assessment. The response frame of the PHQ-9 was thus used for assessing both depressive and burnout symptoms. Mean SMBM and PHQ-9 scores were computed for each participant. The SMBM and the PHQ-9 were each followed by a 7-point item (from 1 for *not at all* to 7 for *totally*) aimed at assessing the extent to which participants attributed their potential burnout and depressive symptoms to work (“If you checked off any of the aforementioned problems, to which extent do you consider these problems to be caused by your work?”).

#### BP

The Borderline Personality Questionnaire (BPQ; Cronbach's α = 0.93; Poreh et al., 2006) was employed to assess BP traits. The BPQ includes 80 dichotomous (“True [1]/False [0]”) items divided into nine subscales, namely, impulsivity, affective instability, abandonment, relationships, self-image, suicide/self-mutilation, emptiness, intense anger, and quasi-psychotic states (for the complete item list, see Poreh et al., 2006, pp. 256–258). These nine subscales reflect the nine diagnostic criteria for BP disorder indexed in the DSM-5 (APA, 2013) and hence allow the investigator to comprehensively cover the features of BP. In a study that compared four different self-report measures of BP traits in relation to the BP module of the Structured Clinical Interview for DSM-IV Axis II Disorders (Chanen et al., 2008), the BPQ was found to have the best mix of characteristics in terms of specificity, sensitivity, positive predictive value, and negative predictive value. Moreover, by comparison with the other three instruments under scrutiny, the BPQ showed the highest overall diagnostic accuracy, a substantially higher kappa with the criterion diagnosis, the highest test-retest reliability, and the highest internal consistency. The BPQ has been used in several recent studies that adopted a dimensional approach to BP (e.g., Fonseca-Pedrero et al., 2011; Gill and Warburton, 2014). For each participant, a mean score was computed for each of the nine subscales of the BPQ. The global BPQ score (ranging from 0 to 1) of each participant was the mean of the mean scores obtained on each subscale.

As suggested by Poreh et al. (2006), we examined the factorial structure of the BPQ, using principal component analysis (PCA). We conducted two independent PCAs, one with the female subsample, and another with the male subsample. In both cases, we used a promax rotation. As recommended by Fabrigar and Wegener (2012) and Salgado et al. (2013), we used multiple decision rules to define the number of components to be rotated. Scree test, parallel analysis, and Kaiser-Meyer-Olkin criterion converged to suggest that two components should be rotated in both sub-samples. The two components explained 53.9 and 55.6% of the overall variance in the BPQ, in the female and the male subsample, respectively (Supplementary Material 1). The two PCAs revealed considerable agreement regarding the scale loadings in the respective principal components, with the exception of the quasi-psychotic states scale. The largest loadings in the first component were for affective instability, abandonment, relationships, self-image, and emptiness. In the second component, the largest loadings were for impulsiveness, suicide/self-mutilation, and intense anger. Based on the loadings and the content of the scales, we termed the first component “affective insecurity” and the second component “impulsiveness.” We used these two components in our analyses of the relationships between BP features and our other variables of interest.

#### Neuroticism

Neuroticism was assessed with a dedicated subscale of the NEO Five-Factor Inventory (NEO-FFI; Cronbach's α = 0.86; Rolland et al., 1998). The neuroticism subscale of the NEO-FFI consists of 12 items (e.g., “I often feel tense and jittery”). Participants responded using a 5-point scale (from 0 for *strongly disagree* to 4 for *strongly agree*). Mean neuroticism scores were computed for each participant. The NEO-FFI is an instrument of reference for the measurement of “Big Five” factors (McCrae and Costa, 2004).

#### Life satisfaction

A questionnaire dealing with life satisfaction was included in the survey to offer participants the possibility of adopting a potentially “positive” perspective on their existence—a possibility that is often perceived as a breathing space by participants requested to complete surveys dominated by “negatively-loaded” items. Satisfaction with life was assessed using the Satisfaction with Life Scale (SLS; Cronbach's α = 0.89; Diener et al., 1985; Blais et al., 1989). The SLS contains five items (e.g., “If I could live my life over, I would change almost nothing”). Items are scored from 1 for *strongly disagree* to 7 for *strongly agree*. Mean SLS scores were computed for each participant. The SLS constitutes a time-saving, psychometrically sound measure of subjective well-being (Pavot et al., 1991).

#### Occupational stress

A short version of the Effort-Reward Imbalance Questionnaire (ERIQ-SV) was used to assess occupational stress (Niedhammer et al., 2000; Siegrist et al., 2009). The ERIQ-SV is comprised of three effort-related items (e.g., “I have constant time pressure due to a heavy work load”; Cronbach's α = 0.72) and seven reward-related items (e.g., “I receive the respect I deserve from my superior or a respective relevant person”; Cronbach's α = 0.73). The assessment of occupational stress relies on the computation of the ratio between job-related efforts and rewards. Mathematically speaking, an effort/reward ratio of 1 reflects perfect balance between efforts and rewards at work. A ratio < 1 suggests that the job gives more than it takes whereas a ratio > 1 suggests that the job takes more than it gives. The effort-reward imbalance model is one of the most prominent models of work stress today (Wang et al., 2012). The ERIQ-SV was intended to provide us with additional information on the relationship between BP and occupational adversity.

#### Sociodemographic and health questionnaire

Participants were asked about their age, sex, current involvement in a conjugal-romantic relationship, and history of depressive disorders. History of depressive disorders was investigated using the following item: “Have you ever been diagnosed for a depressive episode by a health professional (for instance, a family physician, a psychologist, a psychiatrist)? Answer ‘Yes’ only if this diagnosis has resulted in psychotherapeutic treatment and/or treatment with medication.” The second part of the item was intended to limit the risk of false-positive report.

### Data analyses

Both dimensional and categorical analyses were conducted. We mainly relied on correlation analysis, frequency analysis, multiple regression analysis, and Student's *t*-test with Bonferroni correction. For the purpose of our categorical analyses of BP, participants were divided into low and high BPQ scorers. Low scorers were defined by a BPQ global score < 0.50 whereas high scorers were defined by a BPQ global score ≥ 0.50^2^. Regarding the two items related to the attributions of burnout and depressive symptoms to work (1–7 rating scale), we considered participants to markedly attribute their symptoms to work if their scores were ≥5. Such a cutpoint demarcates the upper end of the attributional continuum.

## Results

The raw correlations among the main study variables are displayed in Table 1. BP was moderately associated with burnout and depressive symptoms and strongly associated with neuroticism. Burnout and depressive symptoms more strongly correlated with the “affective insecurity” component of BP than with its “impulsiveness” component. Effort-reward ratios were way above 1 in both women (1.57) and men (1.47), suggesting that, on average, participants viewed their work as stressful. A history of depressive disorders was reported by 32.3% of women and 29.3% of men in our study sample.

**Table 1:** Means *(M)*, standard deviations *(SD)*, Cronbach's alphas (α), and raw correlations among the main study variables.

		***M***	***SD***	**α**	**1**	**2**	**3**	**4**	**5**	**6**	**7**	**8**	**9**	**10**	**11**	***M***	***SD***	**α**
1.	Burnout symptoms (0–3)	0.99	0.70	0.95	–	0.86	0.51	0.48	0.34	0.53	−0.35	0.54	0.31	−*0.00*	*0.01*	0.82	0.75	0.96
2.	Depressive symptoms (0–3)	0.88	0.61	0.86	0.82	–	0.57	0.56	0.34	0.59	−0.43	0.49	0.30	*0.06*	*0.04*	0.73	0.63	0.88
3.	Borderline personality (0–1)	0.22	0.15	0.93	0.51	0.58	–	0.94	0.73	0.75	−0.57	0.33	0.38	*0.07*	−*0.03*	0.19	0.15	0.93
4.	Affective insecurity	0.00	1.00	0.88	0.50	0.56	0.96	–	0.45	0.74	−0.62	0.34	0.36	−*0.09*	−*0.02*	0.00	1.00	0.88
5.	Impulsiveness	0.00	1.00	0.79	0.32	0.37	0.65	0.43	–	0.46	−0.25	0.19	0.28	−*0.01*	−*0.06*	0.00	1.00	0.84
6.	Neuroticism (0–4)	1.92	0.78	0.86	0.55	0.60	0.77	0.76	0.44	–	−0.48	0.37	0.41	*0.03*	−*0.01*	1.61	0.76	0.85
7.	Satisfaction with life (1–7)	4.72	1.31	0.89	−0.41	−0.49	−0.53	−0.54	−0.26	−0.50	–	−0.38	−0.24	−*0.11*	0.13	4.76	1.35	0.90
8.	Occupational stress	1.57	0.58	N/A	0.51	0.50	0.38	0.37	0.20	0.35	−0.34	–	0.29	*0.07*	*0.12*	1.47	0.64	N/A
9.	History of depressive disorders (0/1)	0.32	0.47	N/A	0.17	0.19	0.31	0.25	0.33	0.28	−0.16	0.11	–	*0.10*	*0.04*	0.29	0.46	N/A
10.	Age (in years)	42.21	9.73	N/A	*0.01*	*0.01*	−*0.05*	*0.05*	*0.06*	−0.09	−*0.06*	*0.03*	0.14	–	−*0.02*	46.12	9.10	N/A
11.	Conjugal-romantic relationship (0/1)	0.82	0.39	N/A	−0.07	−0.10	−0.12	−0.14	−0.07	−*0.04*	0.25	*0.02*	−0.07	−0.08	–	0.91	0.29	N/A

*Entries below the diagonal represent women's results (n = 941); entries above the diagonal represent men's results (n = 222). Sex was coded 0 for “female” and 1 for “male.” Italicized correlation coefficients are non-significant (p ≥ 0.05). History of depressive disorders and conjugal/romantic relationship were coded 0 for “absence” and 1 for “presence.” N/A, not applicable. In order to compute the internal reliability coefficients of “affective insecurity” and “impulsiveness” (the two components of the Borderline Personality Questionnaire), we used McDonald's (1985) formula (p. 217)*.

Multiple regression analyses, in which neuroticism and history of depressive disorders were controlled, showed that BP was associated with burnout and depressive symptoms in both women and men (Table 2). More detailed analyses, which distinguished between the two components of BP, revealed that burnout and depressive symptoms were associated with both “affective insecurity” and “impulsiveness” in women. In men, only the associations with “affective insecurity” reached statistical significance. Multicollinearity levels were acceptable, with all tolerance indices >0.40 (see Field, 2009, pp. 223–224).

**Table 2:** Multiple regression analyses with burnout and depressive symptoms as outcome variables.

	**Female sample (*****n*** = **941)**				**Male sample (*****n*** = **222)**			
	**Burnout symptoms**		**Depressive symptoms**		**Burnout symptoms**		**Depressive symptoms**	
	**β**	***p***	**β**	***p***	**β**	***p***	**β**	***p***
**MODEL 1**								
Neuroticism	0.38	<0.001	0.38	<0.001	0.32	<0.001	0.36	<0.001
History of depressive disorders	−0.00	ns	−0.01	ns	0.09	ns	0.05	ns
Borderline personality	0.23	<0.001	0.29	<0.001	0.24	<0.01	0.28	<0.01
Adjusted *R*^2^	0.32		0.39		0.31		0.37	
**MODEL 2**								
Neuroticism	0.37	<0.001	0.37	<0.001	0.36	<0.001	0.37	<0.001
Affective insecurity	0.19	<0.001	0.23	<0.001	0.17	<0.05	0.26	<0.01
Impulsiveness	0.07	<0.05	0.10	<0.001	0.10	ns	0.05	ns
Adjusted *R*^2^	0.32		0.39		0.30		0.37	

*History of depressive disorders was not included in Model 2 given its absence of predictive value in Model 1. ns, non-significant (p ≥ 0.05)*.

By comparison with women displaying relatively low scores on the BPQ, women displaying relatively high scores on the BPQ reported more burnout symptoms, more depressive symptoms, more neuroticism, less satisfaction with life, a more unfavorable effort/reward ratio, and a weaker likelihood to be involved in a conjugal-romantic relationship (Table 3). Similar results were found among male participants, with two exceptions. First, men with relatively low scores on the BPQ and men with relatively high scores on the BPQ did not differ from each other in terms of involvement in a conjugal-romantic relationship. Second, antecedents of depressive disorders were more frequently reported by male high scorers than by male low scorers (Table 3). Most effect sizes were large (Cohen's *d*s ≥ 0.80).

**Table 3:** Comparisons^a^ between participants with relatively low vs. high scores on the Borderline Personality Questionnaire (BPQ).

	**Female sample (*****n*** = **941)**				**Male sample (*****n*** = **222)**			
	**Low BPQ scores (*n* = 903)**	**High BPQ scores (*n* = 38)**	***p*-value**	**Cohen's *d***	**Low BPQ scores (*n* = 213)**	**High BPQ scores (*n* = 9)**	***p*-value**	**Cohen's *d***
	***M* (*SD*)**	***M* (*SD*)**			***M* (*SD*)**	***M* (*SD*)**		
Burnout symptoms	0.95 (0.67)	1.94 (0.72)	<0.001	1.42	0.78 (0.73)	1.75 (0.71)	<0.001	1.35
Depressive symptoms	0.85 (0.57)	1.81 (0.68)	<0.001	1.53	0.69 (0.59)	1.81 (0.61)	<0.001	1.87
Neuroticism	1.88 (0.75)	3.06 (0.63)	<0.001	1.70	1.55 (0.71)	3.01 (0.34)	<0.001	2.62
Satisfaction with life	4.79 (1.27)	3.09 (1.29)	<0.001	1.33	4.85 (1.29)	2.53 (0.84)	<0.001	2.13
Occupational stress	1.55 (0.57)	2.03 (0.57)	<0.001	0.84	1.44 (0.61)	2.19 (0.88)	<0.001	0.99
History of depressive disorders	0.31 (0.47)	0.53 (0.51)	ns^b^	0.45	0.26 (0.44)	0.89 (0.33)	<0.001	1.62
Age	42.20 (9.69)	42.66 (10.68)	ns^b^	0.05	46.02 (9.06)	48.56 (10.36)	ns^b^	0.26
Conjugal-romantic relationship	0.83 (0.38)	0.58 (0.50)	<0.001	0.56	0.90 (0.30)	1.00 (0.00)	ns^b^	0.47

a *Student's t-test with Bonferroni correction was employed for between-group comparisons. Because eight comparisons were carried out, Bonferroni correction resulted in a significance threshold lowered to 0.00625*.

b *ns, non-significant*.

Burnout and depressive symptoms showed very strong raw correlations (*r* = 0.82 in women; *r* = 0.86 in men). When corrected for attenuation, the correlations between burnout and depressive symptoms were nearly perfect (0.91 and 0.94 in women and men, respectively). The “affective-cognitive” symptoms of depression and the “somatic” symptoms of depression showed raw correlations of 0.68 and 0.73 in women and men, respectively. The raw correlation between burnout and the “affective-cognitive” symptoms of depression was 0.70 in women and 0.79 in men. The raw correlation between burnout and the “somatic” symptoms of depression was 0.79 in women and 0.81 in men. The patterns of correlation of burnout and depressive symptoms with the main study variables were very similar, both in women and in men (Table 1).

A little more than half of women and men markedly attributed their burnout symptoms to their job (score ≥ 5). Similar results were obtained regarding the attribution of depressive symptoms to work (50.8 and 50.2% in women and men, respectively; see Table 4 for a detailed view of these results). Very strong correlations were found between the attribution of burnout symptoms to work and the attribution of depressive symptoms to work, *r* = 0.87 and *r* = 0.91, respectively in women and men. BP appeared to be associated with the tendency to (a) attribute one's burnout symptoms to work, *r* = 0.18 among both women and men, and (b) attribute one's depressive symptoms to work, *r* = 0.20 and *r* = 0.25, in women and men, respectively. In women, the attribution of burnout symptoms to work was moderately associated with the severity of burnout symptoms, *r* = 0.54. A similar relationship was found between the attribution of depressive symptoms to work and the severity of depressive symptoms, *r* = 0.48. In men, the attribution of burnout symptoms to work was also found to be moderately associated with the severity of burnout symptoms, *r* = 0.56. A similar relationship was observed between the attribution of depressive symptoms to work and the severity of depressive symptoms, *r* = 0.45.

**Table 4:** Responses to the two items related to the attributions of burnout and depressive symptoms to work.

	**Female sample**					
	**Burnout symptoms attributed to work (*****M*** = **4.50;** ***SD*** = **2.02)**			**Depressive symptoms attributed to work (*****M*** = **4.29;** ***SD*** = **1.93)**		
**Score**	**Frequency**	**Percent**	**Cumulative percent**	**Frequency**	**Percent**	**Cumulative percent**
1	90	10.3	10.3	92	10.1	10.1
2	110	12.6	22.9	121	13.3	23.5
3	85	9.7	32.6	111	12.2	35.7
4	103	11.8	44.4	123	13.5	49.2
5	142	16.3	60.7	162	17.8	67.1
6	157	18.0	78.7	163	18.0	85.0
7	186	21.3	100.0	136	15.0	100.0
Total	873	100.0	–	908	100.0	–
	**Male sample**					
	**Burnout symptoms attributed to work (*****M*** = **4.40;** ***SD*** = **2.16)**			**Depressive symptoms attributed to work (*****M*** = **4.24;** ***SD*** = **2.09)**		
1	28	14.2	14.2	27	13.0	13.0
2	27	13.7	27.9	32	15.5	28.5
3	16	8.1	36.0	21	10.1	38.6
4	17	8.6	44.7	23	11.1	49.8
5	28	14.2	58.9	32	15.5	65.2
6	38	19.3	78.2	33	15.9	81.2
7	43	21.8	100.0	39	18.8	100.0
Total	197	100.0	–	207	100.0	–

The two items (an item related to burnout symptoms and an item related to depressive symptoms) were each associated with a 7-point rating scale, from 1 for “not at all” to 7 for “totally.”

## Discussion

The main objective of this study was to examine the association of burnout with BP traits. Because burnout has been found to overlap with depression, parallel analyses of burnout and depression were conducted. In addition, we carried out sex-specific analyses to offer a more detailed view of the results.

As hypothesized, burnout was found to be associated with BP traits. This association remained statistically significant when controlling for neuroticism and history of depressive disorders. These findings suggest that the impact of BP on individuals' (work) life is not solely accounted for by a general propensity to negative emotionality. In women, burnout was linked to both the “affective insecurity” and the “impulsiveness” component of BP. In men, the link between burnout and “affective insecurity” reached statistical significance whereas the link between burnout and “impulsiveness” did not. These divergent results, however, may reflect differences in sample sizes and statistical power rather than actual between-sex differences. This being mentioned, the features of BP might have different effects in social contexts (e.g., occupational contexts) depending on whether they are displayed by men or by women (Evers et al., 2005; Brescoll and Uhlmann, 2008; Banzhaf et al., 2012). More research on this issue should be conducted. In addition, BP traits were found to be associated with occupational stress, as indexed by effort-reward ratios. These findings are consistent with the view that individuals with BP disorder tend to construct a more threatening and stressful reality of their life in general, and of their work in particular, than individuals with no disorder (Jovev and Jackson, 2006; Baer et al., 2012; Mitchell et al., 2014).

The strong correlations that we observed between BP and neuroticism are consistent with the results obtained by Samuel and Widiger (2008) in a meta-analytic review of the relationships between the five-factor model of personality (FFM) and DSM-IV-TR (APA, 2000) personality disorders. In these authors' study, BP disorder was found to be positively associated with (all facets of) neuroticism. The close connection between BP and neuroticism coheres with the view that DSM personality disorders represent maladaptive or extreme variants of the traits described in the FFM (Trull et al., 2003; Samuel and Widiger, 2008; Few et al., 2014).

The present study informs the issue of burnout-depression overlap in several respects. Because this issue is directly relevant to the interpretation of the link between burnout and BP traits, we address it in detail. First, we found nearly perfect disattenuated correlations between burnout and depressive symptoms (0.91 and 0.94 in women and men, respectively), suggestive of between-construct empirical redundancy (Le et al., 2010; Shaffer et al., 2016). Correlations of such magnitudes are likely to be found when two measures of the same construct are analyzed. As an illustration, Titov et al. (2011), relying on a sample of 172 participants, found raw correlations of 0.72 and 0.73 between the PHQ-9 and the Beck Depression Inventory-II (BDI-II) at two different measurement points. In a similar vein, in a study by Kung et al. (2013), the PHQ-9 and the BDI-II showed raw correlations of 0.67 in a sample of 338 inpatients and 0.81 in a sample of 287 outpatients. The PHQ-9 and the depression subscale of the Hospital Anxiety and Depression Scale exhibited a raw correlation of 0.78 in a 456-participant study conducted by Reddy et al. (2010). Wojciechowski et al. (2000) found raw correlations ranging from 0.70 to 0.77 between the Zung Self-Rating Depression Scale and the depression subscale of the Symptom Checklist-90 at four different time-points (89 ≤ *n*s ≤ 128). Finally, Shirom and Melamed (2006), examining two different groups of professionals, found raw correlations of 0.74 (*n* = 198) and 0.79 (*n* = 236) between the SMBM and another measure of burnout symptoms, the MBI-General Survey (see also Demerouti et al., 2003; Qiao and Schaufeli, 2011).

Second, our results indicated that the association between the “affective-cognitive” symptoms of depression and the “somatic” symptoms of depression, which form a unified entity (depression), was *weaker* than the burnout-depression association (see also Bianchi et al., 2016a)^3^. This finding sheds a different light on the idea that burnout and depression cannot be viewed as a unified entity because of their appreciable, but limited, shared variance (e.g., Schaufeli and Enzmann, 1998, pp. 39–40). Given that the “affective-cognitive” and “somatic” symptoms of depression are considered to form a unified entity with raw correlations around 0.70, there is no apparent obstacle to viewing burnout and depression as a unified entity with raw correlations around 0.80. Such results additionally suggest that, even if our correlations between burnout and depression were somewhat affected by the action of common method variance (CMV; Podsakoff et al., 2003; Spector, 2006), this would be no argument against burnout-depression overlap. Indeed, there is no reason to think that the action of CMV would more strongly affect the correlation between burnout and depression than the correlation between the “affective-cognitive” and “somatic” symptoms of depression.

Third, burnout and depressive symptoms showed similar patterns of association with our other variables of interest, including BP, neuroticism, life satisfaction, occupational stress, and antecedents of depressive disorders. Put differently, in our study, burnout and depression were not distinguishable in terms of nomological network (Le et al., 2010; see also Bianchi and Schonfeld, 2016). In meta-analytic studies, neuroticism has been linked to both depression (Jeronimus et al., 2016) and burnout (Swider and Zimmerman, 2010). Langelaan et al. (2006) concluded that “neuroticism is of prime importance for burnout” (p. 530). Our study suggests that neuroticism is as important vis-à-vis burnout as it is vis-à-vis depression. Life satisfaction has been associated with burnout and depression in many past studies, with degrees of association depending on how the three variables were conceptualized and operationalized (Brand et al., 2010; Erdogan et al., 2012; Hakanen and Schaufeli, 2012). Finally, consistent with our results, Schonfeld and Bianchi (2016) found that (a) burnout and depression correlated to a similar extent with job adversity and (b) individuals scoring at the upper end of the burnout continuum more often presented with a history of depressive (and anxiety) disorders than individuals reporting “minimal” levels of burnout symptoms.

Fourth, we found no clear evidence that burnout symptoms are more strongly attributed to work than depressive symptoms—consistent with the above-mentioned finding that burnout and depression were similarly associated with occupational stress. Remarkably, many participants did not attribute their burnout symptoms to work. These results are of great importance given that the job-induced character of burnout is critical to the identity of the construct and has been claimed to distinguish burnout from depression (Schaufeli and Enzmann, 1998; Maslach et al., 2001; Shirom, 2005). Our results further call into question the involvement of work in the symptoms assessed by burnout measures (Bianchi and Brisson, 2017; Bianchi et al., 2017c; Hakanen and Bakker, 2017).

All in all, these findings support the view that burnout overlaps with depression (Taris, 2006; Ahola et al., 2014; Bianchi et al., 2017a,b,f). Construct proliferation—a violation of the scientific canon of parsimony—has become a major problem in psychology (Schmidt, 2010), particularly in work and organizational psychology (Le et al., 2010; Cole et al., 2012; Allen et al., 2014; Newman and Harrison, 2015; Fogarty and Perera, 2016; Shaffer et al., 2016; Taris et al., 2017). We recommend that greater attention be paid to this problem that threatens theory building and transdisciplinary communication (Bianchi et al., 2017d). As a reminder, the initial development of the burnout construct was not clinically-grounded, nosologically-framed, theory-driven, or comprehensively informed by stress and depression research (Schaufeli and Enzmann, 1998; Maslach et al., 2001; Bianchi et al., 2017e,f). With this background in mind, the lack of discriminant validity of the burnout construct documented today is probably not surprising.

At least three limitations to our study should be mentioned. First, our study was cross-sectional. Therefore, no causal inference can be drawn regarding the links between our variables of interest. Second, we only focused on educational staff. Studies of other occupational groups may help assess the generalizability of our results. Third, we did not focus on BP disorder in the present study (i.e., on BP as a diagnostic entity). It would be useful to investigate BP concomitantly at dimensional and diagnostic levels in the future.

In conclusion, the present study suggests that burnout is associated with BP traits through burnout-depression overlap. Burnout/depressive symptoms may mediate the relationship between BP and variables such as unemployment, job turnover, and work disability. The impact of BP traits in work contexts needs to be further investigated, particularly in longitudinal studies, so that individuals with BP traits can be better accompanied in their occupational life.

## Author contributions

The first author designed the study, collected the data, and wrote a first version of the manuscript. All authors took part in data analyses and reviewed/edited several versions of the manuscript until a consensus was reached on the final version of the paper.

### Conflict of interest statement

The authors declare that the research was conducted in the absence of any commercial or financial relationships that could be construed as a potential conflict of interest.

## Footnotes

^1^A recent systematic review of the literature dedicated to the association of BP disorder with fibromyalgia and chronic fatigue syndrome—two conditions that can be considered close to burnout—concluded that more research is required to clarify possible associations between the three entities (Penfold et al., 2016).

^2^Given that BPQ global scores can range from 0 to 1, a cutpoint of 0.50 conveniently separates participants with relatively low BPQ scores from participants with relatively high BPQ scores. It is worth underscoring, however, that this cutpoint is only used for indicative purposes. This cutpoint has not been clinically validated and should not be mistaken for a “case finder” or viewed as a diagnostic indicator. It follows that no conclusion regarding the prevalence of BP, for instance, should be drawn based on such a cutpoint.

^3^In the study by Bianchi et al. (2016a), the seventh item of the PHQ-9 (“Trouble concentrating on things, such as reading the newspaper or watching television”) was included in the “affective-cognitive” subscale of the PHQ-9, not in the “somatic” subscale of the PHQ-9. Whether this item should be included in the “affective-cognitive” or the “somatic” subscale of the PHQ-9 is open to question given that concentration impairment can be linked to both “somatic” (e.g., fatigue and loss of energy) and “affective-cognitive” (e.g., rumination) phenomena. This being mentioned, when reconducting our analyses using Bianchi et al.'s (2016a) subscale definition, we obtained similar results, calling for the same conclusions.
