# Supplementary material for: Burnout, Depression, and Borderline Personality: A 1,163-Participant Study
Source: Front Psychol. 2018 Jan 11;8:2336. doi: 10.3389/fpsyg.2017.02336 (PMC5769336; doi:10.3389/fpsyg.2017.02336)
Supplement: Supplementary file 1 [file Table1.DOCX]

| **Supplementary Material 1.** Pattern matrices of the Borderline Personality Questionnaire for the female and male samples (principal component analysis with promax rotation). | | | | | |
| --- | --- | --- | --- | --- | --- |
|  | Female sample (*n* = 941) | |  | Male sample (*n* = 222) | |
| Subscale | Component 1 | Component 2 |  | Component 1 | Component 2 |
| Impulsivity | -.07 | **.84** |  | -.27 | **.95** |
| Affective instability | **.68** | .21 |  | **.63** | .29 |
| Abandonment | **.85** | -.10 |  | **.57** | .25 |
| Relationships | **.79** | -.16 |  | **.68** | .02 |
| Self-image | **.79** | -.02 |  | **.91** | -.15 |
| Suicide/Self-mutilation | -.03 | **.72** |  | .34 | **.37** |
| Emptiness | **.81** | .04 |  | **.93** | -.12 |
| Intense anger | .36 | **.38** |  | .35 | **.50** |
| Quasi-psychotic states | **.40** | .08 |  | .18 | **.35** |
| Initial eigenvalue | 3.80 | 1.05 |  | 3.99 | 1.01 |
| Percentage of variance | 42.21 | 11.65 |  | 44.33 | 11.26 |
| *Note.* Larger loadings appear in bold. | | | | | |
